# Microplastics and nanoplastics: Exposure and toxicological effects require important analysis considerations

**DOI:** 10.1016/j.heliyon.2024.e32261

**Published:** 2024-05-31

**Authors:** Emmanouil D. Tsochatzis, Helen Gika, Georgios Theodoridis, Niki Maragou, Nikolaos Thomaidis, Milena Corredig

**Affiliations:** a Department of Food Science, CiFOOD, Centre for Innovative Foods, Agro Food Park 48, Aarhus N, 8200, Denmark; b FoodOmicsGR Research Infrastructure, AUTh Node, Center for Interdisciplinary Research and Innovation (CIRI-AUTH), Balkan Center B1.4, 10th Km Thessaloniki-Thermi Rd, P.O. Box 8318, GR 57001, Thessaloniki, Greece; c Biomic AUTh, Center for Interdisciplinary Research and Innovation (CIRI-AUTH), Balkan Center B1.4, 10th Km Thessaloniki-Thermi Rd, P.O. Box 8318, GR 57001, Thessaloniki, Greece; d School of Medicine, Aristotle University of Thessaloniki, 54124, Thessaloniki, Greece; e Department of Chemistry, Aristotle University of Thessaloniki, 54124, Thessaloniki, Greece; f Laboratory of Analytical Chemistry, Department of Chemistry, National and Kapodistrian University of Athens, 15771, Athens, Greece

**Keywords:** Degradation of MPs/NPs, Oligomers, Exposure to MPs/NPs, Analysis, Toxicological considerations, Risk assessment

## Abstract

Microplastics (MPs) and nanoplastics (NPs) pervade both the environment and the food chain, originating from the degradation of plastic materials from various sources. Their ubiquitous presence raises concerns for ecosystem safety, as well as the health of animals and humans. While evidence suggests their infiltration into mammalian and human tissues and their association with several diseases, the precise toxicological effects remain elusive and require further investigation. MPs and NPs sample preparation and analytical methods are quite scattered without harmonized strategies to exist at the moment.

A significant challenge lies in the limited availability of methods for the chemical characterization and quantification of these contaminants. MPs and NPs can undergo further degradation, driven by abiotic or biotic factors, resulting in the formation of cyclic or linear oligomers. These oligomers can serve as indicative markers for the presence or exposure to MPs and NPs. Moreover, recent finding concerning the aggregation of oligomers to form NPs, makes their analysis as markers very important.

Recent advancements have led to the development of sensitive and robust analytical methods for identifying and (semi)quantifying these oligomers in environmental, food, and biological samples. These methods offer a valuable complementary approach for determining the presence of MPs and NPs and assessing their risk to human health and the environment.

## Highlights

• Measuring and assessing MPs and NPs based on oligomers presence.
• Degradation of MPs, NPs into smaller oligomers, monomers.
• Oligomers and monomers analysis: insights, implications.
• Toxicological consideration: MPs/NPs and oligomers in human body.
• Human health and risk assessment of microplastics and nanoplastics.

## 1 Introduction

The production of plastic materials continues to increase, their use is widespread as well as their presence in the environment. Application and use of plastic material has large variety; products can range from food contact materials (FCM) and packaging, to automotive, electrical equipment and to consumer products. These products can be in the form of flexible robust plastics, films, plastic bags, trays, cups and containers, but also coatings, labels and glues [1,2].

Small fragments of plastic material are ubiquitous in the environment, in foods and in drinking water, and have become a source of increasing concern worldwide. These particulate pollutants, defined as microplastics (MPs) or nanoplastics (NPs), depending on their size, have a clear, long term impact on the environment, on human and ecosystem health [[3], [4], [5]]. MPs are considered the plastic particles with a size ranging between 1 μm up to 5 mm [6,7], without ever presenting a well-defined lower size limit, with a variety of reported sized and dimensions [7]. “*MPs*” is therefore an ill-defined term employed for plastic particles, for which no official or established definition exists at the moment and furthermore with no clear size or shape discrimination [5,7]. “*NPs*” may refer to submicron particles, ranging between 1 and 1000 nm, usually lower than 100 nm, as it has been proposed for nanoparticles in the nanotechnology field [5,7]. Finally, as recently reported and based on their respective size, oligomers of certain plastics (e.g. polyethylene terephthalate; PET, polybutylene terephthalate; PBT, polystyrene; PS) can also be considered as NPs, since their dimensions are few nanometres (1–3 nm) [5]. The human exposure to MPs and NPs is considered as certain, through various environmental channels including water and food [3,7,8]. Apart from MPs and NPs, plastic debris poses a threat to the biota mainly due to three types of effects: direct contact with plastic particles, the release of co-transported pollutants, and the leaching of backbone oligomers or additives [9,10].

It has been proposed that a part of the ingested particles, and especially NPs or oligomers, can pass through the gastrointestinal system [11,12] without so-ever knowing the involved pathways or mechanisms. In any case, it is reported that the smallest the NPs the more possible this passage to occur. However, the overall number of studies is still very limited. *In vitro* studies, applying model particles often use extremely high concentrations and in vivo studies provide weak results, which do not allow a mechanistic evaluation that withstands critical considerations [8]. Thus, there is a real challenge in establishing dose–responses to be integrated in risk assessment studies. No molecular, metabolic alterations or initiating events can be stated, and these mechanisms are important to identify adverse effects. Overall, addressing the challenges associated with long-term exposure to MPs and NPs requires comprehensive analytical strategies, especially in relation to accumulation and potential leaching of chemicals (e.g., additives, plasticizers etc.) as well as formed oligomers. These chemicals might have adverse effects on various organ systems, potentially leading to possible chronic health issues and therefore literature reports the need for long-term and chronic studies which at the moment are limited [8].

Hence, overall, the risk assessment of both MPs and NPs is still not possible, due to the current lack of data in relation to occurrence, exposure, biodistribution and clear linkages to resulting effects. The importance of obtaining answers to these gaps is increasing and is a challenge for scientists involved in all fields of human, food and environmental safety. The present review scope is to present different aspects related to the effects rising from MPs and NPs in relation to the formation and release of their degradation products (oligomers) as source of NPs but also as markers. Hence, the main goal is the connection between release of oligomers and presence of MPs and NPs that can support any assessment of potential impact on human health. For the latter, existing analytical methods should be adapted and validated for MPs and especially for NPs. This is more critical in particular for their determination, identification and quantification in complex matrices such as food and environmental matrices, which at the moment are of high research priority [8], but also feed, soil and other complex environmental samples.

## 2 Formation of MPs/NPs

**Fig. 1:**
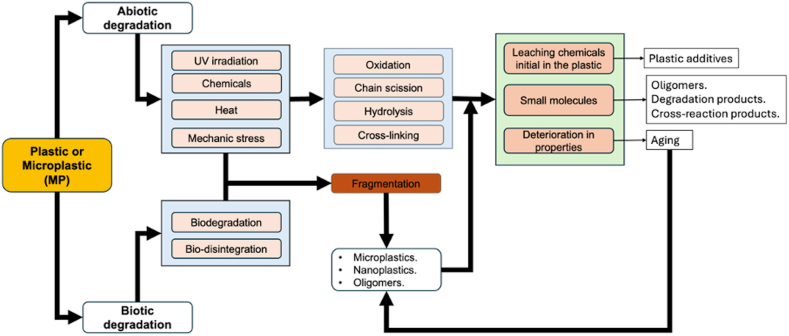
General processes contributing to the degradation of plastics.

A recent review [13] highlighted the potential formation of MPs in the environment together with a clear perspective of the plastic degradation processes. The abiotic degradation might involve several environmental factors such as light, temperature, water, air and mechanical forces [13]. In addition to abiotic degradation, biotic degradation of plastics can also occur by organisms. These organisms can degrade plastics either physically by biting, chewing or digestive fragmentation or biologically by biochemical processes, such as enzymatic activity or microbiological activity [13]. Especially in case of biotic degradation, hydrolytic and oxidative degradation reaction of plastics can be generated by various extracellular enzymes, resulting in the scission of the polymer chain, producing short-chain polymers and small molecular polymer fragments (e.g., oligomers, dimers, and monomers) [14]. These degradation products are also widespread in the environment and could potentially be absorbed by humans and animals [15]. Small molecular degradation chemical products can be integrated and be subject to intracellular metabolism [13,15] (Fig. 1).

(adapted from Ref. [13]).

In addition, for certain plastics, such as polystyrene (PS) the respective monomer has been investigated for potential toxicity, carcinogenicity and genotoxicity [16,17]. Also in case of PS, as it is valid for other plastics, oligomers might be also present as side-reaction products and they shall be also considered [18].

## 3 Degradation of MPs/NPs to oligomers

Although the degradation of plastic is a general problem for all plastic materials, very few studies focus on their breakdown. It shall be noted that certain of the generated oligomers are of Cramer III toxicity class [19], according to Quantitative structure–activity relationship (QSAR) models [5,20,21]. Moreover, the European Food Safety Authority (EFSA) recently published a Guidance on harmonized methodologies for human health, animal health and ecological risk assessment of combined exposure to multiple chemicals [22]. In this case, the comprehensive characterization of chemical mixture composition is necessary in order to be associated with any potential effects [22]. Although complete toxicological studies are not available at the moment, the next section will delve into a few case studies, recently published in the literature regarding MPs degradation to oligomers, that will serve as examples of the current state-of-the-art.

### 3.1 Polystyrene (PS) and polyethylene (PE)

**Fig. 2:**
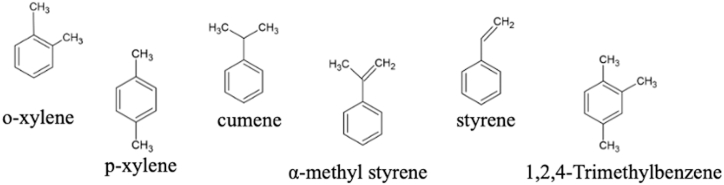
PS degradation products.

In a recent study, Zhu et al., while trying to develop a methodology for PS MPs removal from water, identified several factors that can contribute to their degradation, such as the occurrence of hydrothermal degradation reactions. The main degradation products reported in this work were low-molar mass polystyrene (PS) oligomers as well as styrene monomers (α-methyl styrene, styrene) or styrene or-benzene-based compounds (as o-xylene, cumene, and 1,2,4-trimethylbenzene) as reported in Fig. 2 [23].

(adopted from Ref. [23])

**Fig. 3:**
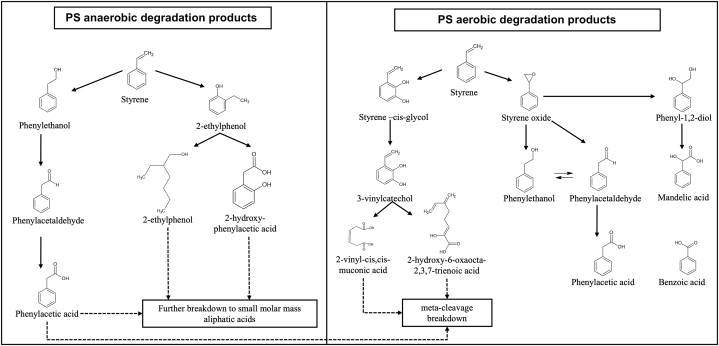
PS in vivo formation of degrading compounds after ingestion.

Identical to Zhu et al. [23], another study reported and highlighted (bio)chemical pathways for the biodegradation of PS and the formation of styrene monomers, PS oligomers (dimers, trimers) and styrene-related compounds (e.g. phenylacetic acid, styrene-glycol, phenyl-1,2-diol) (Fig. 3). Two main metabolic pathways for the biodegradation of styrene have been reported in relation to aerobic and anaerobic degradation [24].

(adapted from Ref. [24]).

Additionally, it was recently shown that PS can transform not only into MPs and NPs particles, but also can degrade further into basic structural styrene oligomers (SOs) units, such as 2,4-diphenyl-1-butene (styrene dimer) and 2,4,6-triphenyl-1-hexene (styrene trimer), by decomposing at 30 °C. From a structural perspective, certain oligomers can have dimensions higher than 1 nm and below 100 nm (e.g. PET and PS oligomers). Hence, they can be classified as NPs, based on the existing definitions [25].

Low-density polyethylene (LDPE) also can be degraded abiotically, as demonstrated in a long-term accelerated weathering experiment [26]. LDPE material is prone to abrasion, which manifests initially with surface cracking. A large number of secondary particles with a high degree of crystallinity are formed, with sizes down to the nm scale. These particles can consist of highly polar oligomers i.e. carboxylic acids and alcohols, leading to their agglomeration [26].

In addition to the degradation results mentioned above, it has also been reported that both PS and polyethylene (PE) might also undergo a random internal scission (or endo-type depolymerisation) in the environment during biodegradation [27].

A study on the fate of PE and PS plastics during gastrointestinal transit in the human body has been recently reported. Krasucka et al. performed an *in vitro,* mechanistic study. It was shown that MPs particles passing through are surface modified by digestion, generating more hydrophobic surfaces, and generating new nano structures (as identified by electron microscopy) that may lead to an increase of adsorption of other organic contaminants once released again in the environment [28]. Moreover, for MPs from the major plastic polymers, might undergo UV-radiation and oxidative degradation that could result in the formation of oxygen-bearing moieties, particularly carboxylic acid groups [29,30]. These modified compounds can be considered to be at the “nanoparticles" or "nanoplastics” size, whilst seem to be prone to be absorbed and translocated in intestinal epithelial cells [24], raising the level of urgency in filling the knowledge gap in this area.

### 3.2 Polyethylene terephthalate (PET)

For polyethylene terephthalate (PET), it has been shown that degradation occurs, eventually resulting in the formation and release of cyclic or linear oligomers from the original polymer [14,20,21]. PET oligomers can be prone to hydrolysis, even at low temperatures (4 °C) [31], indicating the potential to degrade or be transformed to molecules of even smaller molar size and mass.

Formation of oligomers occurs from plastics even if they are considered as oligomer-free [32]. It has been demonstrated that cyclic oligomers progressively accumulate in molten plastic throughout the processing stages, eventually stabilizing at a plateau. An analysis of the fraction of the re-formed cyclic oligomers showed that the majority of cyclic trimer (60–70 %) was found at once the reaction reached a maximum equilibrium plateau. Before the establishment of this equilibrium, an unusual behaviour was observed in the relative proportion of cyclic trimer and tetramer during the first steps of their formation [32].

Studies on the fate of PET MPs in the gastrointestinal digestion system are also starting to become available. Tamargo et al. reported a structural degradation of the PET MPs by colonic microbiota. Although it is known that PET is resistant to biodegradation, there are bacterial enzymes, i.e. cutinases, lipases, carboxylesterases, and esterases that could degrade PET to different extents, leading to various degrees of decomposition/depolymerisation [33], and eventually to the formation and leaching of monomer or oligomers. Similarly, it has also been reported that during biotic degradation (biodegradation) of a PET plastic, an organism can release exo-enzymes such as laccase from *Staphylococcus epidermis* [34,35] and PETase from *Ideonella sakaiensis* [36], which can degrade these plastic polymers into oligomers and monomers [34]. Furthermore works have documented the presence of well-defined oligomers and/or low molar mass biogenic species in environmental samples resulting from MPs/NPs degradation [34,37].

**Fig. 4:**
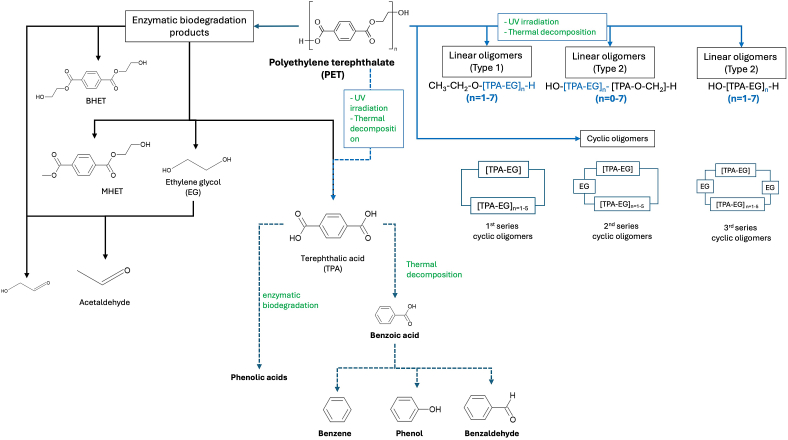
Main degradation products of PET.

Moreover, PET is a common thermoplastic polymer used in various applications, including packaging, textiles, and bottles. Overall, the specific degradation products of PET can vary depending on factors such as temperature, processing conditions and UV irradaiton and the presence of catalysts or contaminants [20,23,[38], [39], [40]]. When PET undergoes degradation, it can produce several by-products, including:

A. Acetaldehyde: This is one of the most common degradation products of PET, especially when exposed to high temperatures. Acetaldehyde can affect the taste and odor of food and beverages stored in PET containers.
B. Carboxylic compounds and alcohols: Various carboxylic acids, such as terephthalic acid (TPA), benzoic acid, ethylene glycol (EG) or phenol, have been reported as potential degradation products. These acids can further react or leach out from the polymer matrix.
C. Oligomers and monomers: PET degradation can result in the formation of oligomers and monomers, including TPA and ethylene glycol. These smaller molecules can potentially migrate out of the polymer matrix, affecting the properties of the material. As recently reported, oligomers of PET might be linear or cyclic. For linear three types are reported, while for cyclic oligomers of PET, three series exist (1st, 2nd and 3rd) depending on the integration of different unit of EG among the TPA-EG repeating units (See Fig. 4).
D. Aromatic compounds: Some aromatic compounds, such as benzene derivatives, may also form as degradation products of PET, especially at high temperatures.

A schematic representation of potential pathways for the formation of degradation products, via main routes (UV irradiation, thermal decomposition or enzymatic biodegradation) are presented in Fig. 4.

(adapted from Refs. [20,23,[38], [39], [40]]).

A recent work for PET revealed that aggregation of oligomers lead to the formation of NPs size particles, where oligomers were identified as a major fraction of the sub-micrometre particles [41]. Therefore, understanding these processes and mechanisms driving the aggregation of oligomers into NPs is crucial for assessing the environmental fate and impact of plastic pollution. Further research is needed to elucidate the mechanisms involved and develop strategies for mitigating NPs pollution in the environment.

### 3.3 Poly lactic acid (PLA)

Compostable bioplastics such as PLA have also been studied for formation of MPs, although data is quite limited. In aqueous media, hydrolysis and cleavage of the ester linkages in the polymeric backbones occur, which might lead to destruction and fragmentation of PLA polymer films into MPs [42]. These hydrolysis products can be further modified (e.g. oxidation) due to oxidative environment as om case of industrial composting. The hydrolysates can be monomers, dimers or oligomers [43]. Similarly, the abiotic hydrolytic degradation of polycaprolactone (PCL) to submicron-plastic sizes (MPs to NPs) and oligomers. It was reported that the formed oligomers from PCL and, to a larger extent NPs (<100 nm), were toxic [10].

**Fig. 5:**
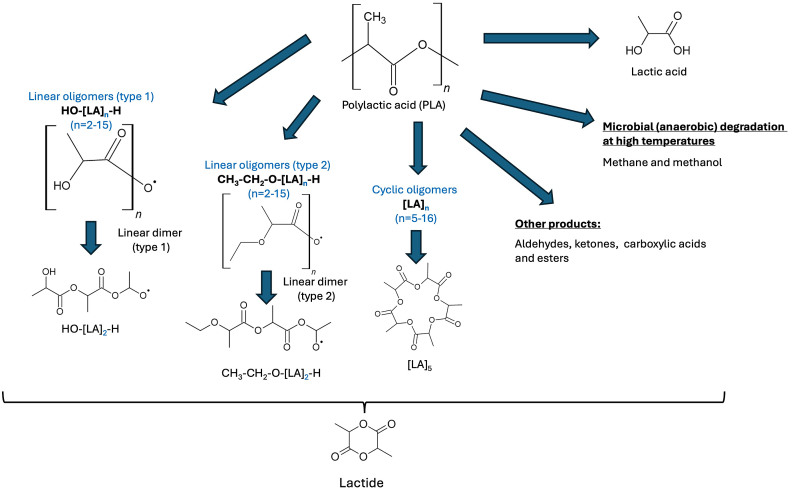
Main degradation products of PLA.

Polylactic acid (PLA) is a biodegradable and bioactive thermoplastic derived from renewable resources. When PLA undergoes degradation, whether through thermal, mechanical, or environmental factors, it can produce several main degradation products, such as lactic Acid, oligomers and monomers (see Fig. 5. In certain cases, other Degradation Products can be generated, depending on the specific degradation conditions, such as aldehydes, ketones, and esters.

(adapted from Refs. [44,45,46]).

For the main degradation products, Lactic acid results from PLA hydrolysis, when exposed to moisture and high temperatures. This process is accelerated under acidic or basic conditions. In case of oligomers and Monomers, these are formed during PLA degradation, breaking into smaller molecular weight chains, forming oligomers and monomers such as lactide [44,45]. For oligomers, different types were identified such as type 1 (HO- … -H linear oligomers) and type 2 (CH_3_–CH_2_- … -H linear oligomers) and cyclic oligomers, with repeating lactic acid units that can vary from 2 up to 16 [45]. From lactic acid monomer or oligomers, either linear or cyclic, lactide can be generated, which eventually can be further polymerized to form PLA or degrade into lactic acid [47]. Moreover, various low molecular weight organic compounds, such as cyclic diesters and carboxylic acids, can form as degradation products of PLA. These compounds can be released as gases or leach out of the polymer matrix. In certain conditions, such as anaerobic environments or high temperatures, PLA degradation can produce methane and methanol as by-products. Other degradation products of PLA, depend on the specific degradation conditions, leading to the generation of compounds like aldehydes, ketones, and esters [46].

## 4 Oral exposure to MPs/NPs

The increasing use of plastic materials leads to the increase of the plastic waste worldwide that can be degraded to smaller particles, such as MPs and NPs and eventually to oligomers or monomers. These particles may enter the human body via different pathways, such as food, drinking water and environment among others. Recent studies bring evidence of the interaction between gastrointestinal microbiota and MPs/NPs, and it is possible to assume that biodegradation activities in human colon will occur with formation of various metabolites, oxidative products, oligomers and reactive monomers.

For MPs, adsorption though the mucosa, and in particular intestinal absorption may be limited due to their size which ranges between 1 and 5 μm, however research in this regard is lacking. It is however important to point out that all these plastic materials contain a mixture of chemicals intentionally added during their manufacture, or non-intentionally added (NIAS), which could migrate through the polymeric matrix into the food, the bolus or the cell mucosa and eventually be absorbed by the gastrointestinal system. However, not enough data exist at the moment that can allow for an accurate and precise assessment of the risks associated with the dietary exposure to MPs/NPs from food. The lack of robust methodologies to study the fate of these molecules from food to the blood circulation is currently one of the main bottlenecks. Furthermore, very limited information on human intake, toxico-kinetics and long term toxicity of these contaminants exist [48].

**Fig. 6:**
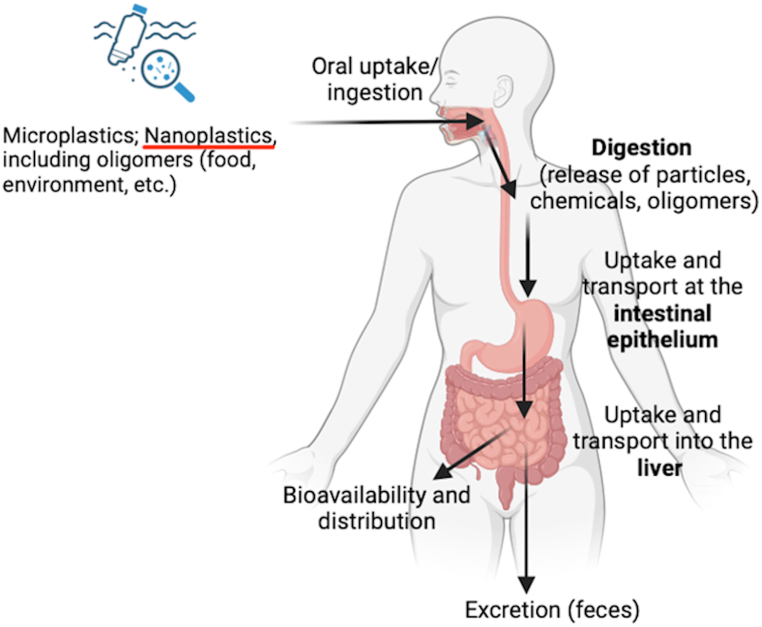
Oral exposure to MPs and NPs.

In regard to oral exposure, MPs and NPs can reach the human body through oral uptake or ingestion, through food but also through the environment. Oral ingestion is followed by a number of steps that can influence the particles per se. The harsh gastric environmental conditions might lead to potential digestion products and reactive species, as well as to certain interactions, including, and not limited to, the contact with digestive fluids, contact to intestinal cells, uptake and transport in the intestine and liver, and finally excretion [8]. These interactions are illustrated in Fig. 6.

(adapted from Ref. [8]; graphic was generated with BioRender software).

Laboratory simulations have demonstrated that plastic MPs and NPs can be generated from the fragmentation and disruption of plastic material [49], or they can just be released from products containing them as functional components, as for example, in textile or cosmetics [50]. Recent research [23] evaluated the fate of MPs during gastrointestinal transit and demonstrated using scanning electron microscopy (SEM), Fourier-Transform infrared spectroscopy (FTIR) and ultra-high performance liquid chromatography-quadruple time-of-flight mass spectrometry (UHPLC-QTOF-MS) that additional structures formed and that new functional groups, mostly hydrophobic and ionic in nature formed, with increased capacity to bind pollutants, and becoming in turn new MPs in the environment. MPs and NPs, after transiting the upper gastrointestinal tract, may be further modified by the gut microbiota. This has been recently demonstrated in insects, which showed the ability to metabolize plastic and degrade it [51,52] when part of their diet, and the degradation was attributed to their gut microbiota [24]. The fate of these particles in more complex organisms has not been studied in detail and more research is needed.

The digestive tract has a very high surface area, due to its absorbing function and provides a potential route for orally ingested particles to enter the body. In pharmaceutical science, many oral delivery formulations are based on polymer nanoparticles [53], as they promptly pass through the intestinal mucosa and protect the active pharmaceutical ingredient from breakdown, by enzymes and acids during transit. Studies on nanoparticles design have resulted in a wealth of knowledge on how to enhance transport through the gastrointestinal barrier, with low risks of inflammation [54]. Particles with sizes larger than 10 mm do not penetrate the mucus layer [53]. The gastrointestinal epithelium is designed to protect us from foreign components. These cells are the last barrier to absorption, they are cylindrical cells connected to each other through tight junctions, with a brush border membrane on top covered by a dense mucus layer. After digestion, MPs or NPs must first diffuse through the mucus layer, and then enter blood circulation. A recent study evaluated via fluorescent labelled model nanoparticles the effect of basic physical parameters such as size, charge and hydrophobicity on the ability to overcome these intestinal barriers [55]. The physical properties of the polymeric material will then have an impact on the bio-adhesion, the interactions with the mucus layer or cell absorption. Particles less than 4 mm interact with the intestinal layer. Factors such as size, negative charge, and hydrophilicity are shown to facilitate the nano and microplastic particles transfer through the mucus layer more easily. Once through the layer of mucus, surface charge or hydrophobicity seem to be improving the process of transintestinal transport [55]. It was demonstrated, by using Caco2 and HT29 MTX cell models, that polymer NPs showed the highest uptake, and that cell uptake was negatively correlated with NP's size [55]. Transcellular transport of the plastic NPs has been related to various mechanisms, such as diffusion, phagocytosis, and endocytosis, by which the particles are transported through the cell and released at the basolateral membrane [[56], [57], [58]]. In other words, the larger particles were more likely to be retained by the mucus layer, however their physical and colloidal properties will affect their transport through the intestinal barrier.

Therefore, once absorbed and transferred into the blood circulation, plastic particles may accumulate in various tissues and organs. However, it is not clear which pathways are involved and what is the fate of these MPs and NPs, nor which are the mechanisms involved for their degradation, including the breakdown of MPs in NPs and further to smaller building units (e.g. monomers, oligomers).

Moreover, the products of degradation of MPs and NPs are of critical importance, which may result in the formation of additional smaller particles [6] that can eventually pass the cell membranes [11]. Especially for oligomers, a recent study by Järvenpää et al. studied the permeation of polyethylene (PE) and bis(2-hydroxyethyl) terephthalate (BHET) PET's polymer building unit, into cells, where they reported for the first time the permeation of plastic oligomers through cell membranes, by mimicking cell membranes and applying Parallel Artificial Membrane Permeability Assay (PAMPA). The results indicated that a passive diffusion for the existing formatted small plastic oligomers occurs and that the interplay with membrane bilayers could be an important transport mechanism into cells [59].

Therefore, based on all the recent and new recent developments, it is strongly indicated that humans are exposed to chemicals resulting from plastic material or from MPs/NPs.

## 5 Other routes of exposure

The primary routes by which MPs and NPs are entering the human body are through ingestion, primarily by oral exposure and through the gastrointestinal tract (GIT). However, other routes that have been identified are via inhalation [60], skin absorption, mainly from personal care products (PCPs) containing them [61] or through contact with surfaces that can attach either MPs or NPs to the skin [62]. Inhalation is emerging as another potential route of exposure to microplastics. Research has found microplastics in indoor and outdoor air samples, indicating that inhalation of airborne particles is possible. While inhalation may not be as significant a route of exposure as ingestion currently, it is gaining attention as scientists explore the potential health impacts of microplastics in the air, as recently reported by a literature review [60] and a recent report from the European Commission [63]. Following entry into the body, MPs and NPs have the potential to migrate from the initial organ of exposure to other parts of the body [61]. However, a through recent reviews highlighted that based on the current understanding, it was concluded that a dermal translocation of MPs or NPs is rather unlikely [61].

## 6 Toxicological considerations

PS-NPs are more likely to enter the cells than the PS's respective micron-sized PS-MPs. Furthermore, the intake is proportional to the exposure time of the tested human digestive tract cells. It was also demonstrated that the nano-PS enters the cells more easily than micro-PS. The 0.1–5 μm PS showed low cytotoxicity to CCD841CoN and HIEC-6 cells although the PS-MPs caused significant higher cell membrane damage compared to the respective PS-NPs [64]. In this area, much more work has been carried out within the domain of pulmonary toxicology. An *in vitro* assessment of polyethylene terephthalate nanoplastic, on pulmonary tissue indicated a decrease of mitochondrial membrane potential with the increasing nano-PET exposure, consistent with the change of reactive oxygen species, a major mechanism of toxic effect [65]. Another study with PS-NPs instead induced neutrophil extracellular traps (NET) formation, with involvement of reactive oxygen species, peptidyl arginine deiminase 4 (PAD4), and neutrophil elastase [66].

Yip et al. evaluated the effect of the presence of three different nanoparticles materials, such as polymethyl methacrylate (PMMA), polystyrene (PS) and polyvinyl chloride (PVC) on marine organisms (spawned barnacle nauplii), their associated toxicity and the presence of respective oligomers. The presence of low molecular weight oligomers such as dimers, trimers and tetramers were observed in all tested spawned barnacle nauplii [67]. Furthermore, the injection of the oligomers (which are nanosized) of the respective plastics was shown to lead to apoptosis and gut and metabolic alterations [24,51,52,68]. Exposure of Zebrafish to PS-MPs (3–12 μm) indicated stress indices, including DNA damage, lipid peroxidation, autophagy, ubiquitin levels, caspases activation and certain metabolic effects [69].

Concerning effects of MPs and NPs in the liver, it is reported that MPs and NPs that enter the human body, can be transported to various organs, including the liver, and eventually pose a hepatotoxic effects [70,71]. Hence, once in the liver, MPs/NPs may trigger inflammatory responses, oxidative stress, and disruption of cellular functions, leading to potential liver damage and dysfunction. Current research suggests that microplastics and nanoplastics can accumulate in the liver over time, potentially causing long-term harm. Additionally, these particles may interact with other toxins or chemicals present in the body, exacerbating their toxic effects on the liver [70,71]. However, the quantification and the effects of this accumulation cannot be assessed, due to the lack of proper analytical methodologies.

It has been shown that both MPs and NPs can lead to an imbalance of the gut microbiome flora, which is exhibiting a reduction of beneficial bacteria and a respective increase of harmful bacteria [72]. The latter effect might have consequences on the intestinal barrier, and certain bacterial products or species might enter blood circulation with potential risks to other tissues and organs [72]. The intestinal walls’ breakdown could potentially be related to some extent to a “*leaky gut syndrome*” effect, which would in turn allow harmful bacteria and substances to escape into the bloodstream. Conversely, the presence of low levels of MPs and NPs in food may be more harmful to individuals with irritable bowel disorders, due to their leaky gut conditions, compared to healthy individuals.

Humans are exposed to MPs and NPs mainly through environment mainly by ingestion or through other routes, as mentioned before. In relation to human health, MPs and NPs toxicity can include oxidative stress, inflammatory lesions, and then increased internalization or translocation through tissues. An important drawback is the limited existing analytical arsenal available to assess MPs and NPs content in various substances and materials. However, it is expected that small-sized NPs, such as oligomers, are those with a superior rate of uptake and translocation through tissues [60], but this hypothesis has yet to be demonstrated, most probably due to the lack of proper analytical methodologies.

## 7 Recent advances in analysis

### 7.1 Sample preparation

For sample preparation of MPs and NPs, several techniques are reported in the literature [[73], [74], [75], [76]], such as (1) chemical digestion with acids, enzymes or other chemicals to dissolve organic matter and extract microplastic; (2) Separation by physical methods, involving separation based on density, size (filtration) or ultrasonic; (3) Oxidative digestion, using oxidizing agents, like H_2_O_2_ to break down organic matter and facilitate the separation of MPs; (4) Extraction solvents to extract MPs or NPs, applying even pressurized or accelerated solvent extraction.

The use of strong acids or oxidizing agents in digestion procedures can potentially break down larger plastic particles into smaller fragments, including oligomers, thereby altering the original composition of the sample [[73], [74], [75], [76]]. Mechanical agitation during the application of physical processes may lead to the fragmentation of plastic particles, producing smaller particles and potentially oligomers. Filtration processes may subject microplastics to mechanical stress, leading to fragmentation and the generation of smaller particles, including oligomers. Ultrasonic treatment is often employed to disperse microplastics from aggregated samples or to facilitate the extraction of microplastics from complex matrices. However, ultrasonic energy can induce physical and chemical changes in plastics, potentially resulting in the formation of oligomers. Moreover, prolonged exposure to oxidative conditions can induce degradation of plastics, leading to the formation of oligomers as by-products. Enzymatic digestion involves the use of enzymes to break down organic matter and release microplastics from biological samples like feces or tissues. While enzymatic methods are generally gentler compared to chemical digestion, certain enzymes may catalyze the breakdown of plastics into oligomers under specific conditions. Solvent extraction may alter the chemical composition of plastics and promote the release of oligomers, particularly if the solvents have reactive properties [[73], [74], [75], [76]].

**Fig. 7:**
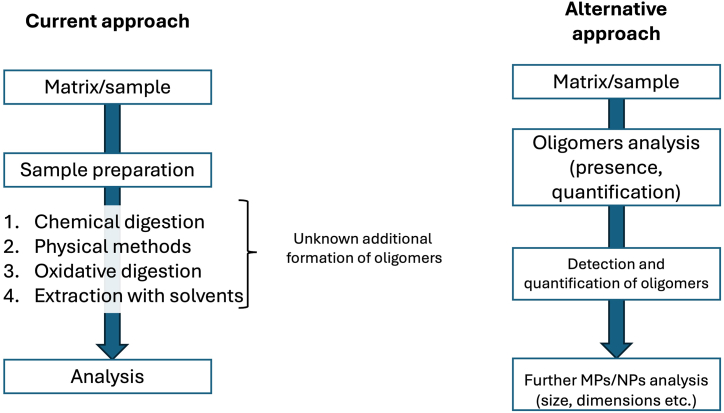
Sample preparation techniques for analysis of oligomers. Current and alternative proposed approach.

The degradation of MPs or NPs, by the sample preparation technique applying current analytical strategies (Fig. 7), can be already considered as a drawback, especially using hyphenated techniques (e.g. Pyr-GC/MS), which is the only technique that can clearly discriminate plastics [12]. The formation of oligomers, which are structurally similar to polymers, can lead to higher signals and their identification as MPs. Hence, the choice of sample preparation technique can significantly influence the formation of these oligomers during MPs and NPs analysis. Thus, careful consideration of the method's impact on plastic degradation and oligomer formation is essential, in order to ensure accurate and reliable results [[73], [74], [75], [76]], whilst providing information for their formation.

Based on the existing knowledge, in this review we would like to report a new approach, measuring MPs and NPs based on initial identification of oligomers presence (see Fig. 7). The latter poses a novel approach to detection, by targeting oligomers rather than all MPs and NPs per se, applying comprehensive analytical strategies and workflows. As already discussed, oligomers are intermediate molecules formed during the degradation of plastics, researchers can potentially assess the extent of plastic degradation and exposure in environmental samples. In some cases, oligomers can be considered a priori and based on the current definition as NPs (>1 nm), and therefore they can be used for exposure and potential toxicity assessments. Analytical techniques such as mass spectrometry, chromatography, and spectroscopy can be utilized for this purpose. By detecting and quantifying oligomers, researchers can indirectly estimate the abundance of MPs and NPs in the environment, after verifying though their presence due to leaching.

It is important to note that measuring MPs based solely on the presence of oligomers may have limitations. Oligomers can originate from various sources besides plastic degradation, and their presence may not always correlate directly with MPs abundance. Additionally, oligomers may degrade further into smaller molecules or undergo transformation processes in the environment, making their detection challenging, for which important analytical developments already exist and are reported [2].

For MPs and NPs, here lacks a universally accepted standard or protocol for sample preparation, especially concerning NPs. The choice of method relies heavily on the sample's characteristics and the subsequent analytical approach [74]. The evolving nature of plastics might contribute another layer of complexity to both sample preparation and analysis, a challenge we explore further in this discussion. It cannot be omitted, especially for NPs, that can be formed due to aggregation of oligomers based on not well-known mechanism. This outcome makes NPs analysis, discrimination and classification more challenging and demanding, considering the current lack of analytical methods [41]. Thus, in this case it can be overlooked the overestimation of NPs, since they refer mainly to oligomers.

### 7.2 Analysis

Analyzing MPs and NPs involves distinct methodologies due to differences in their size ranges. Overall, while there are similarities in the analysis of MPs and NPs, differences in size range necessitate distinct methodologies tailored to each particle size category. NPs may require more sophisticated quantification methods due to their smaller size and potential aggregation behaviour, especially as it was recently reported for oligomers [41]. Additionally, surface charge, chemical composition, and aggregation state are important characteristics to consider when characterizing NPs. In addition, the latter consideration was confirmed by a recent report from the European Commission, where it was clearly stated that detecting NPs in samples remains a growing field. While existing analytical techniques may be effective for the detection of MPs, they struggle to identify and discriminate particles smaller than 5 μm. Further research is highly needed in respect to the development of reliable methods for characterizing plastic particles and in the low μm and nm range [63].

Current analytical methods such as spectroscopy, molecular imaging, hyphenated techniques combined with mass spectrometry such as time-of-flight mass spectrometry (TOF-MS), pyrolysis with GC-MS, light scattering or microscopic techniques have been proposed to selectively detect MPs and NPs in complex matrices such as food products [77], although no clear discrimination occurred for MPs and NPs and the complexity of the matrix poses substantial challenges. Nevertheless, very recently, plastic-derived particles at the nano and microscale have been identified in human blood [12], human lung tissue [78], placenta [79] as well as PET oligomers in blood [11], although without a clear description nor extrapolation of the involved pathways. Overall, analysis of biological samples for additives, monomers, and oligomers existing in plastic materials can be associated with exposure providing valuable insights into human exposure to MPs and NPs, and their potential health risks. Continued research is essential to better understand the toxicity of these plastic-associated chemicals and develop strategies to mitigate human exposure to MPs and NPs.

Furthermore, human health risk assessment requires data on the chemical identity and concentration levels of these micro- or nano-sized structures leaching in the environment, in food samples and drinking water as well as in biological samples. Their composite nature makes it particularly challenging. Hence more work is needed to develop harmonized methods to detect and quantify the MPs/NPs in environmental, food and biological samples.

In case of polyvinyl chloride (PVC), PET, PE or fluorescent-labelled PVC in oyster and fish tissue, Chang et al. applied an enzymatic digestion with Corolase7089 followed by lipase to digest most of the organic tissue with high efficiency and then analysed with Raman spectroscopy and Nanoparticle Tracking Analysis (NTA) the residues. Results presented good recoveries for PVC and PET [80].

Another approach for the determination of MPs/NPs in aqueous samples was proposed by Zhou et al. by applying cloud-point extraction to preconcentrate traces-of NPs, and in combination with thermal treatment, to then analyse the samples by pyrolysis gas chromatography (Pyr-GC/MS) at a temperature of 590 °C using a HP-5MS (30 m × 0.25 mm, 0.25 μm) column. The study showed that the analyte styrene is not indicative for PS identification and quantification in environmental samples, although it is an abundant PS-pyrolysis product. In contrast, styrene trimer (*m/z* 312) is specific for PS identification; the common ion fragment for styrene trimer with the strongest signal intensity, *m/z 91*, was selected as the quantitation ion [81].

Although, Pyr-GC/MS can be applied for reliable identification and quantification of MPs/NPs without limitations of particle sizes, the extraction and isolation from biological matrices can be considered as challenging. This methodological approach was tested by Zhou et al. for quantitative analysis of PS and PMMA NPs in tissues of aquatic animals. The animal tissue was primarily dissolved with alkaline treatment with sodium hydroxide (NaOH) and tetramethylammonium hydroxide (TMAH) followed by an extraction with organic solvent for separation and isolation of the NPs, screening respective pyrolytic products, such as styrene, 3-butene-1,3-diyldibenzene (styrene dimer) and 5-hexene-1,3,5-triylbezne (styrene trimer) for PS plastic, methyl methacrylate for PMMA nanoplastic [82]. However, by analysing the molecular markers, technically, the MPs/NPs are not quantified, but everything coming from the MPs/NPs (oligomers included).

An accurate approach to determine the presence of MPs/NPs consists in the depolymerisation of the plastic particles to their structural compounds and then quantify them via a hyphenated technique. Wang et al. reported for the first time this semi-quantitative and indirect approach for the quantification of polycarbonate (PC) and PET MPs in environmental samples by liquid chromatography−tandem mass spectrometry (LC-MS/MS) and after alkali-assisted thermal hydrolysis/depolymerisation to polymers building blocks, such as bisphenol A (BPA), bisphenol di-glycidyl ether (BADGE) and terephthalic acid. The semi-quantification was performed using isotope-labelled internal standards, namely ^13^C-BPA and d_4_-PTA, respectively [65,83]. In this context, Tian et al. developed a catalytic depolymerisation of PET with ethylene glycol, for the quantification of PET MPs and NPs in environmental matrices. They applied a reaction at high temperature (190 °C) for the formation of bis(2-hydroxyethyl) terephthalate (BHET), which is the PET monomer. The analysis was performed by LC-MS/MS for the analysis of d PET MPs in environmental samples using this semi-quantitative approach. However it shall be pointed out that many oligomers might exist in PET [20]. Thus in this case as in case of Zhou et al. [82], not only the MPs/NPs are quantified but everything coming from PET (oligomers included). An identical approach was applied by Castelvetro et al. for nylon 6 and nylon 6,6 (polyamide; PA) and PET quantification in wastewater treatment plant sludge. The sample preparation involved extraction of the samples, acidic hydrolysis with strong acid (HCl), neutralisation of the sample and derivatization with fluorophore compounds for PA, while for PET the sample was quantified based on terephthalic acid. PA was analysed with high pressure liquid chromatography coupled fluorescence detection (HPLC-FLD) while PET analysed with HPLC with ultraviolet detector (UV) [84]. Analysing the components will therefore result in an accurate estimation of the presence of various plastic components, and ideally, this approach should be combined with a physical technique estimating the presence of MPs or NPs.

Bai et al. [85] recently reviewed an overview of the existing methodologies used for the MPs analysis in foods, which represent a complex matrix, with additional complications when trying to identify microparticles. To date, researchers select FT-IR and Raman spectroscopic tools, including also μ-Raman and μ-FTIR imaging, for MPs determination. However, these techniques can be quite resource intensive. For sample preparation digestion of organic material was the most frequently preferred technique, using appropriate solvents. Other techniques, such as Attenuated Total Reflection Mid-Infrared (ATR-MIR), Energy Dispersive X-ray (EDX), hot needle, Laser scanning confocal microscopy (LSCM), Scanning electron microscopy (SEM), SEM and scanning electron microscope and energy-dispersive X-ray spectroscopy (SEM-EDX) and Pyr-GC/MS have been applied for MPs analysis in food samples to a lesser extent [85]. Corti et al. reported methodology for analytical characterization and quantification of MPs and NPs in environmental samples, using a one-dimensional (1D) and two-dimensional (2D) nuclear magnetic resonance (NMR) spectroscopy [37].

Similarly, to what reported above, other reports [67,68] presented similar strategies for extraction and detection of microplastics in foods and marine systems, with enzymatic digestion followed by hyperspectral imaging, SEM and thermo-analytical methods being promising techniques [67]. In addition, microwave-assisted extraction (MAE) after acid digestion, may be a simple, fast and accurate methodology to prepare the samples, for a range of different types of MPs in food and food waste, and could be used in combination with attenuated total reflectance Fourier-transform infrared spectroscopy (ATR-FTIR) [86]. However, it is clear that there is an urgent need for standard or harmonized protocols [87]. It is important to point out that for the chemical digestion, it is reported that the presence of high concentrations of digestive agents such as acids (HNO_3_ and HCl), bases (NaOH and KOH), and oxidative agents (H_2_O_2_) can lead to further degradation or changes of MPs after digestion [43].

For the latter, when MPs are ingested by organisms, whether in the environment or through food consumption by humans or other animals, changes can occur during digestion. These changes can affect the physical and chemical properties of the microplastics, potentially influencing their toxicity, bioavailability, and environmental fate, such as (1) Size Reduction, surface modifications, chemical alterations, cross-interaction with biological tissues or/and fluids, release of additives or plasticizers, formation of NIAS, formation of reactive oxygen species (ROS) and finally facilitating their translocation and spread to other organs or may be excreted from the organism in feces. These changes in microplastics following digestion can have implications for both environmental and human health. Understanding the fate and effects of digested microplastics is essential for assessing their ecological impacts and developing appropriate mitigation strategies [43,[88], [89], [90], [91]].

Despite these challenges, measuring MPs based on oligomers can provide valuable insights into plastic pollution and degradation processes, as well as to the assessment of exposure. This approach may complement existing methods for microplastic detection and contribute to a more comprehensive understanding of plastic pollution in the environment. Further research is needed to validate and refine this approach for accurate microplastic quantification.

## 8 Conclusions

Microplastics (MPs) and nanoplastics (NPs) pervade our environment and food supply. Without accurate quantification, conducting risk assessments for food or environmental exposure becomes exceedingly challenging. Both fossil-based and biodegradable plastics can degrade into smaller MPs and NPs, potentially posing health risks. The mode of human exposure to these particles remains debatable, particularly concerning their presence in food, necessitating further evaluation. Assessing oral exposure to MPs and NPs through food is intrinsically linked to understanding their fate in the gastrointestinal tract, where interactions with bacteria and enzymes occur. These particles may break down into smaller fragments or transform into harmful chemicals (oligomers) capable of reaching human organs or the bloodstream.

Analytical challenges hinder the quantification of MPs and NPs for exposure risk assessment. Recent advancements suggest utilizing identified oligomers from plastics as marker compounds for exposure assessment, coupled with physical techniques like spectral imaging analysis using FTIR and micro-Raman spectroscopy. However, these techniques require harsh sample preparation methods that could inadvertently alter the MPs and NPs being studied. Presently, there are no standardized methods for determining the concentrations and chemical profiles of MPs and NPs. Urgent efforts are needed to develop harmonized and validated methods to advance research in this field based on proper and carefully design sample preparation techniques.

## Disclaimer

The manuscript does not represent nor meant to represent an EFSA (European Food Safety Authority) opinion.

## Funding

This research did not receive any specific grant from funding agencies in the public, commercial, or not-for-profit sectors.

## CRediT authorship contribution statement

**Emmanouil D. Tsochatzis:** Writing – review & editing, Writing – original draft, Visualization, Validation, Supervision, Resources, Project administration, Methodology, Investigation, Formal analysis, Data curation, Conceptualization. **Helen Gika:** Writing – original draft, Investigation, Formal analysis. **Georgios Theodoridis:** Writing – review & editing, Writing – original draft, Visualization, Investigation, Formal analysis, Conceptualization. **Niki Maragou:** Writing – review & editing, Writing – original draft, Visualization. **Nikolaos Thomaidis:** Writing – review & editing, Writing – original draft, Visualization, Methodology, Conceptualization. **Milena Corredig:** Writing – review & editing, Writing – original draft, Visualization, Validation, Resources, Project administration, Methodology, Funding acquisition, Formal analysis, Data curation, Conceptualization.

## Declaration of competing interest

The authors declare that they have no known competing financial interests or personal relationships that could have appeared to influence the work reported in this paper.
